# Large Area Nano-transfer Printing of Sub-50-nm Metal Nanostructures Using Low-cost Semi-flexible Hybrid Templates

**DOI:** 10.1186/s11671-016-1346-4

**Published:** 2016-03-15

**Authors:** Robin D. Nagel, Tobias Haeberle, Morten Schmidt, Paolo Lugli, Giuseppe Scarpa

**Affiliations:** Institute for Nanoelectronics, Technische Universität München, Theresienstr. 90, München, 80333 Germany

**Keywords:** Nano-transfer printing, Thin-film, Nanofabrication, Material science, Nanotechnology

## Abstract

In this work, we present a method for printing metal micro- and nanopatterns down to sub-50-nm feature sizes using replicated, defect-tolerant stamps made out of OrmoStamp®; material. The relevant parameters for a successful transfer over large areas were investigated and yields above 99 % have been achieved. Comparing our results to conventional nano-transfer printing using PDMS stamps, we find that the more rigid hybrid polymer used here prevents unintended transfer from interspaces between structures of large distance due to roof collapse and deformation of nano-sized structures due to lateral collapse. Yet, our stamps are flexible enough to ensure intimate contact with the underlying substrate over large areas even in the presence of defect particles. Additionally, the presented patterning technique is resist-, solvent-, and chemical-free and is therefore ideally suited for applications in organic nanoelectronics where standard nanostructuring methods can harm or destroy the organic material.

**Electronic supplementary material:**

The online version of this article (doi:10.1186/s11671-016-1346-4) contains supplementary material, which is available to authorized users.

## Background

Nano-transfer printing (nTP) is a promising technique to directly produce patterns of metallic films at the nano-scale on different kinds of substrates without the need of conventional lithographic methods. Here, the pattern is initially defined on a stamp as a 3D-relief structure. After metal deposition on the entire stamp area, its relief is brought into intimate contact with the target substrate. If the adhesion of the metal film towards the substrate is stronger than towards the stamp, the film will adhere on the target substrate upon release of the stamp [1, 2]. Consequently, nano-scale metal structures can be easily defined in a purely additive process. As this process does not necessarily involve high temperatures, nor any form of chemistry (e.g., organic solvents, developer solutions, etc.), it is especially interesting for applications where harsh conditions should be avoided (e.g., organic electronics) [3]. Note, that in a nTP process, while the stamp itself needs to be structured with some sort of high-end, cost-intensive lithographic methods (e.g., e-beam lithography), as it then serves as a template for multiple transfer processes, the initial effort is capitalized more efficiently. The quality of this transfer printing process depends mainly on the quality of the stamp, the difference in adhesion between stamp/metal and substrate/metal, and the metal film itself [4, 5]. Silicon stamps, where the structure is defined by partially etching a silicon wafer to produce a 3D-relief structure have proven to be suitable for nTP. The silicon surface can be modified by a self-assembled monolayer (most prominently perfluorooctyltrichlorosilane [PFOTS]) to reduce its surface free energy which significantly reduces the adhesion of metals [6]. We previously demonstrated the fabrication of arrays of nano-scale tunneling diodes using nTP with structured silicon stamps [7].

Nonetheless, there are a few drawbacks involved with using silicon stamps: due to the high elastic modulus of silicon, such stamps can in general not easily adapt to inhomogeneous surfaces or particles on the substrate. As a result, the reliability of the transfer process is limited. Additionally, remaining metal on the stamp can significantly impede the establishment of conformal contact in subsequent transfer attempts and thus prevent a successful transfer of the metal film. As a result, nTP stamps in general can be mostly used only once. Consequently, a better approach is to use the silicon stamp as a master template for replication of a daughter, or working stamp based on a cheaper and preferably more flexible material. Those working stamps would still be one-time-use-only, yet the replication process can be very fast and results in a fresh and clean stamp for each metal transfer.

One prominent example is polydimethysiloxane (PDMS), which has not only been widely adopted for micro-contact printing (*μ*CP) [8, 9], but also for nano-transfer printing. Its inherently low surface free energy enables the transfer of metal films without further modification of the stamp’s surface. Moreover, the low Young’s modulus of PDMS (<100 KPa) [10] facilitates the establishment of intimate contact between stamp and target substrate and allows transfer printing without application of external pressure. Yet, at the same time, this flexibility can also lead to unwanted defects during transfer caused by sagging or collapsing of the 3D structures on the stamp. This limits the minimal achievable structure size and can lead to collapse of high-aspect-ratio features during transfer [11].

Here, we describe a method to use OrmoStamp®;, a commercially available UV-curable, solvent-free, organic/inorganic-hybrid polymer from Micro resist technology GmbH, Berlin, as a viable, cost-efficient material for stamp replication in a nTP process. OrmoStamp®; has initially been developed for UV nanoimprint lithography (UV-NIL) as an alternative to the use of expensive quartz wafers and is suitable for replication of silicon master templates with structures in the low nanometer range [12]. Once cured, the optical transparent replica has a Young’s modulus of 650 MPa (according to manufacturer’s processing guidelines [micro resist technology GmbH]), and its surface can be modified with the same organic chemistry (PFOTS) as silicon to decrease its surface free energy. We demonstrate a successful approach to use OrmoStamp®; working stamps to reliably transfer print at the same time both micrometer- and nanometer-sized metal structures onto silicon target substrates. Furthermore, we investigate the influence of different process parameters on the achievable transfer yield.

## Methods

### OrmoStamp-Based Master Replication

**Fig. 1:**
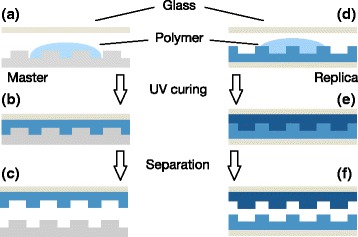
OrmoStamp®; two-fold, adapted UV-NIL replication process. First, a negative replica of the silicon master is casted, which then serves as a template to replicate the positive working stamp

The fabrication of working stamps comprises two successive replication processes (basically two adapted UV-NIL processes): (1) negative replication of silicon master, and (2) replication of the negative replica to obtain a positive working stamp. First, the silicon master is exposed to oxygen plasma and then coated with a self-assembled monolayer (SAM) of PFOTS (perfluorooctyltrichlorosilane) in a desiccator at low pressure and room temperature by physical vapor deposition for 30 min. Afterwards, the master is baked on a hotplate at 150 °C for 30 min to remove any physisorbed molecules. Next, a defined amount of the OrmoStamp®; resin is placed on the master, and a thin glass plate is placed carefully on the droplet (Fig. 1a). To improve the adhesion of the OrmoStamp®; resin to the glass backing, the latter is coated with an adhesion promoter (OrmoPrime08®;) before the replication process. Without applying any pressure, the resin spreads between master and glass to form a thin layer (20–30 *μ*m) and fills up any gaps by capillary forces. When the resin is fully spread, it is exposed to UV light from a mask-aligner (Hg lamp, 350 W, ∼ 7 mW cm^−2^ at 365 nm [i-line]) for 12s to initiate the curing process (Fig. 1b). Afterwards, the glass plate together with the cured polymer can be carefully released from the master by lifting the glass with a thin razor blade. The cured replica stamp requires a hard bake for 30 min at 130 °C on a hot plate (Fig. 1c). Finally, the replica is exposed to a mild oxygen plasma and coated with a PFOTS SAM as described above. We checked the efficacy of this SAM treatment by means of a static water contact angle (CA) measurement: the coated surface exhibits a CA of 110°, while an uncoated surface has a CA of ∼80° (Additional file 1: Figure S1).

We can then use this negative replica to produce a positive working stamp following the same procedure (Fig. 1a–c). The final working stamp is backed by a ∼ 0.1 mm thin microscope cover glass. A thin backing of the working stamp is favorable to retain the flexibility of the polymer layer. This way, we obtain a transparent, semi-flexible, defect-free, identical copy of the original silicon stamp. We are able to reliably produce multiple, identical working stamps from a single negative replica of a silicon master.

### Metal Transfer Printing

**Fig. 2:**
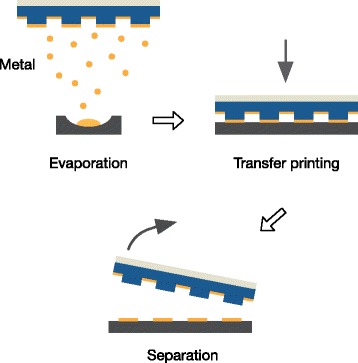
Schematic overview of the transfer printing technique. A metal coated stamp is brought into intimate contact with a receiving substrate. Upon release of the stamp, the metal layer sticks to the target substrate

In this article, we mainly use silicon as a model substrate, due to its importance in semiconductor industry and research. In principle, a variety of substrate materials (glass, plastics, organic polymers, just to name a few) can be used the same way as presented in the following procedure for metal transfer printing with replicated stamps (a nTP carried out on a glass substrate is shown in Fig. 7c: (1) the ready-to-use stamp is coated with a gold/titanium metal layer in a high-vacuum physical vapor deposition system. Typically we use 20 nm gold and 3 nm titanium. The thin titanium layer improves the adhesion of the gold layer to the target substrate (silicon). (2) The target substrate is cleaned (acetone/isopropanol) and preconditioned in an oxygen plasma. (3) The stamp is placed with the metal layer facing downwards onto the target substrate and put into a pressure chamber (Obducat NIL machine). (4) A uniform pressure is applied via compressed nitrogen for a specified period of time. Optionally, the temperature can be increased during the transfer printing process. (5) Finally, the stamp is lifted from the substrate (see Fig. 2). Micrometer-sized structures can be easily transfer printed using OrmoStamp replicas over a large area.

## Results and Discussion

### Wide Gap, Large Area Transfer Print (*μm*-TP)

**Fig. 3:**
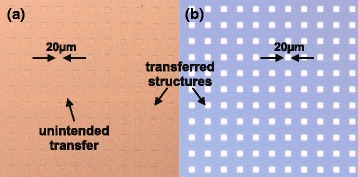
Comparison between transfer print of a Au/Ti thin-film onto a pristine Si substrate with (**a**) a flexible PDMS stamp and (**b**) a semi-flexible polymer stamp. The unintended transfer between the 20 *μ*m metal structures indicates a collapse of the flexible PDMS stamp. The more rigid polymer stamp enables transfer even for structures where *h*<<*w*

We fabricated OrmoStamp®; working stamps on 0.1 mm glass slides with a diameter of 18 mm up to 50 mm from a 3-in silicon master stamp with arrays of different sized rectangles (3 −20 *μ*m edge lengths). The replication process followed the two-fold procedure outlined above. The target substrate was a silicon wafer with native oxide. Due to the flexibility of the thin glass-backed OrmoStamp®; working stamps, uniform and intimate contact is easily established over the entire area of the stamp when sufficient pressure is applied. Squares from 3 to 20 *μ*m edge length were successfully transferred. Simultaneously, the material is rigid enough to prevent collapse of structures which would lead to unintended transfer from interspaces (“roof collapse”) [11]. Note, that the structures of the stamp had a height of only 300 nm with distances of up to 40 *μ*m. Roof collapse is a common defect when using stamps made from other, more elastic materials, e.g., PDMS. To directly compare our working stamps to PDMS stamps, we fabricated PDMS stamps from the same silicon master, evaporated the same amount of metal and attempted to transfer the metal from the PDMS stamps to silicon substrates. We followed well-established protocols for metal transfer with PDMS reported in different publications [1–3, 5, 13]. As can be seen in Fig. 3—even without external pressure—the low structures from the PDMS stamp easily collapse and get in contact with the substrate, thereby causing unintended transfer.

Additionally, we observed that Au/Ti films transferred with OrmoStamp®; working stamps have significantly lower surface roughness (∼ 1 nm) as compared to identical films transferred with PDMS working stamps (∼3–6 nm). The roughness was determined after transfer on the Si substrate by atomic force microscopy (root-mean-square roughness measurements can be found in the Additional file 1: [Figure S2]).

The stability of OrmoStamp®; working stamps together with their uniformity over large areas, enables transfer printing of various structures with challenging aspect ratios and feature sizes. To further demonstrate and investigate the usefulness of this approach, we reduced the feature sizes to the sub-micrometer regime.

### Transfer Print of Nanometer-Sized Structures (nm-TP)

With decreasing feature size towards the nanometer regime, controlling the nTP procedure accurately gets more and more important. Hence, a precise knowledge of the process parameters and their effect on the transfer yield is crucial for implementing nTP as a stable and high-throughput nanostructuring method. In the following, a thorough investigation and optimization of these parameters is performed by a quantitative analysis of transfer-printed Au/Ti-layers in order to be able to evaluate the stability of the nTP method using replicated semi-flexible stamps.

#### Fabrication

In this part of the work, a silicon master stamp with pillar shaped structures of 75 nm in diameter, 150 nm pitch, and 100 nm height was used on a 5 by 5 mm square structured area. This offers the possibility to investigate the nano-transfer print (nTP) process of structures in the sub-100 nm range as well as the homogeneity and stability of the transfer over a comparably large area. According to the procedure stated in Section “Methods”, the master mold was replicated twice in order to obtain a positive working stamp. Silicon wafers with native oxide were used as substrates. Further details on the process can be found in the Additional file 1.

#### Characterization

The quality of nTP can be evaluated by introducing the *yield factor*, which we define as

(1) $$  \text{yield}=\frac{\textrm{number of transferred structures}}{\textrm{total structures on stamp}}\cdot 100\;.  $$

Images of transferred pillars were taken using a SEM (Zeiss NVison40), the number of transferred structures were counted (using the image analysis software *ImageJ*) and related to the total numbers of pillars on the stamp in the corresponding area. For a quantitative analysis, each image consisted of about 2000 pillars, and for each sample, five images in total were taken and evaluated (one in the center and one near to each corner of the squared structured area).

The process parameters printing duration, applied pressure, and temperature during the nTP were chosen to be investigated as well as the pretreatment of substrate and stamp using oxygen plasma activation. A statement about the stability and reproducibility of the process could be made since several samples for each parameter set were evaluated due to the easy working stamp fabrication using OrmoStamp®; technology.

#### Influence of Process Parameters

**Fig. 4:**
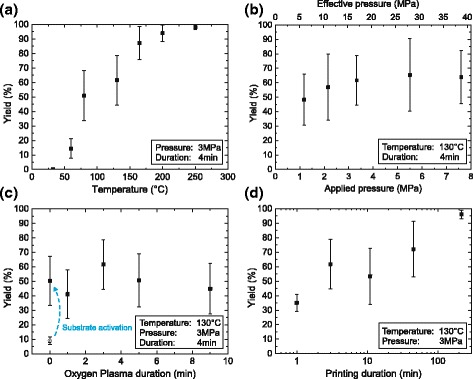
Nano-transfer print of 75 nm Au/Ti metal pillars. The resulting transfer yield is shown as a function of (**a**) temperature during the imprint process, (**b**) applied gas pressure and the calculated effective pressure below the pillar structures, and (**c**) oxygen plasma pretreatment duration of the stamp (while keeping a constant substrate activation duration of 6 min). An attempt with neither substrate nor stamp activation is marked with an *open symbol*, (**d**) duration of the nTP process

In Fig. 4a–d, the yields of the transfer prints while varying the process parameters temperature, applied pressure, oxygen plasma pretreatment, and process duration are shown.

##### Temperature Influence

The dependency of yield on the temperature during the nTP process is given in Fig. 4a. At room temperature, no metal transfer could be observed. It can be seen that transfer of nanopillars with a diameter of 75 nm only occurs above temperatures of about 50–60 °C. Starting around this value, there is a strong improvement of the yield with increasing temperature reaching values above 99 % at 250 °C over the whole transferred area. Thus, temperature is one of the main parameters to improve the nTP process considerably. For common nanoimprint stamps made out of silicon or quartz, high temperatures are not an issue, which is not generally the case for polymer materials. According to the manufacturer’s instructions (micro resist technology GmbH), OrmoStamp®; is chemically and physically stable for nanoimprints up to 160 °C. Although we applied temperatures up to 250 °C in our experiments, we did not identify any changes or damaging of the stamp. However, high temperatures with very long process durations could affect the properties of OrmoStamp®; and should be avoided.

In order to be able to evaluate the influence of other process parameters on the transfer yield, a temperature of 130 °C was chosen in the following since changes in a specific parameter can lead to a measurable shift of the transfer yield in this temperature regime (Fig. 4a). If not explicitly mentioned otherwise, all following variations were carried out in an Obducat nanoimprinter at this temperature with an applied pressure of 3 MPa for 4 min.

##### Pressure

The imprint pressure of the Obducat nanoimprinter was varied between 1.1 and 7.6 MPa which corresponds to an effective pressure underneath the protrusions between 6.0 and 38.7 MPa due to the fill factor of the stamp of about 19.3 *%*. With increasing pressure, only little improvement of the yield can be observed. It can be assumed that a sufficient pressure is mainly needed for the formation of an intimate contact between the Au/Ti film and the silicon substrate. The experiments presented here suggest that a pressure of about 1 MPa already leads to an adequate contact, and it can be assumed that even smaller pressures are feasible. This is a requirement to perform nTP also on unstable substrates and materials, for which high mechanical stresses can be critical.

##### Oxygen Plasma Treatment

An oxygen plasma is used to clean the contact surfaces of substrate and metal film as well as for the generation of hydroxyl groups on the silicon surface, which presumably favor a chemical bond between Si and Ti on elimination of water. In Fig. 4c, the stamp activation duration (while keeping a constant Si-wafer pretreatment) and its influence on the transfer yield is shown. The result suggests that nTP is hardly affected by the plasma treatment of the gold-titan layer. A good transfer yield was possible even without an O_2_-plasma treatment of the stamp. Yet, an activation of the silicon substrate was still required. An attempt without substrate oxygen plasma activation is marked in Fig. 4c with an open symbol. As can be seen, with neither substrate nor stamp activation, the transfer is suppressed, reaching only yield values below 10±3 *%* which is significantly lower than using the default recipe preparation.

Since the nTP process was started immediately after the evaporation of the metal films on the stamp, a sufficiently clean surface can already be expected. Transfer prints using some weeks old, already-metal-coated stamps show considerably lower quality results. However, after applying 3-min oxygen plasma treatment to those coated stamp surfaces right before the nTP, the same yield value as with newly evaporated stamps can be achieved. In contrast, substrate activation was always needed for a high yield.

##### Transfer Print Duration

Transfer prints with different process durations have been investigated (Fig. 4d). Note the logarithmic scale of the time-axis. A significant increase in transfer yield with increasing process time was found. Values comparable to those at raised temperature above 200 °C can be reached applying only 130 °C for a longer time. Yet, the duration of the process needs to be extended to about 2–3 h.

### Sub-50-nm nTP

**Fig. 5:**
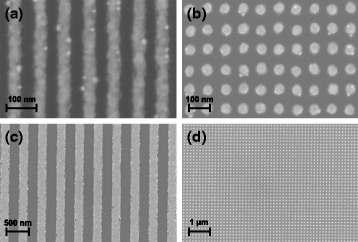
Nano-transfer print of various Au/Ti metal structures on a Si substrate with native oxide. **a** 40-nm line width and 80-nm pitch, **b** 45-nm pillar diameter, 90-nm pitch, **c** 200-nm line width, 400-nm pitch, and (**d**) 75-nm pillar diameter, 150-nm pitch (standard stamp used for process parameters evaluation)

In Fig. 5, SEM images of transferred gold lines and pillars with different dimensions are shown. Optimized process parameters found in Section “Transfer Print of Nanometer-Sized Structures (nm-TP)” (200 °C, 3 MPa applied pressure, 4-min printing time, plasma activation: 3 min stamp/6 min substrate) were used and homogeneous transfer of the entire structured area was obtained. Lines with 40-nm width and 80-nm pitch can be easily achieved, which demonstrates the potential of the nTP process with cheap semi-flexible replicated stamps in the sub-50-nm range. nTP works stable and reproducible, and there is evidence that nTP is only limited by the resolution of the stamp.

The transfer quality can be tuned by varying temperature and printing duration. Although it is possible to reduce the applied temperature when using longer process durations, this is contrary to the advantage of a fast nTP process. However, this is a promising approach for temperature-sensitive substrates and after all faster and cheaper than conventional nanolithography techniques like e-beam lithography.

### Nanostructure Shape Retention

The method presented in this paper includes a two-fold replication of a master mold and additionally a final transfer print. Therefore, it is important to examine how the dimensions of the final shape on the target substrate changes compared to the original one. The shape retention of the nanostructures after the UV-curing step during the replication process is the first critical point. According to the manufacturer, a volume shrinkage of 4–6 % takes place during this step. This is obviously unfavorable for a high lateral dimension accuracy. Using AFM measurements, the nanostructure step-height on the silicon master and on its copies were measured and are summarized in Table 1. As can be seen, the initial height of the silicon master pattern of 94.1 ± 1.6 nm decreases after the first OrmoStamp copy to a value of 90.1 ± 1.1 nm which corresponds to a shrinkage of −4.3 %. The final working stamp has a structure step-height of 87.0 ± 2.4 nm (−3.4 %). The shrinkage of both steps is in good agreement with the expected value of around 4 %. If the working stamps are used for nanoimprint, this height reduction is particularly relevant. For transfer printing this plays a subordinate role, since the height is only important to prevent a metal overgrowth of the structures during evaporation.

**Table 1:** UV-curing induced shrinkage (height)

	Master	Daughter	Working stamp
Height (nm)	94.1 ±1.6	90.1 ± 1.1	87.0 ± 2.4
Shrinkage (nm)		4.0 ± 1.9 (−4.3 %)	3.1 ± 2.6 (−3.4 %)

As the height reduction arises from a volume shrinkage, this will also occur laterally and is more important in the nTP case. However, this can be determined easily and accurately by measuring the pitch of the regular distributed structures averaged over long distances (several micrometers). This was carried out using SEM and the pitch of the master structures was directly compared to the one of the final nTP (Table 2). No measurable difference was found. This can be explained by the glass backing used for the stamps, which prevents a lateral shrinkage of the polymer. If the polymer is applied to softer materials (e.g., plastic films) the strained layer can result in a bulging of the stamp.

**Table 2:** Lateral shrinkage (pitch)

	Master	nTP
Pitch (nm)	151.0 ± 1.5	151.1 ± 1.2
Difference (nm)		0.1 ± 1.9 (0.0 %)

**Fig. 6:**
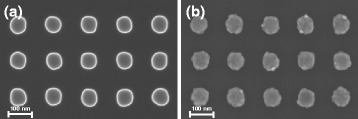
Shape retention characterization. SEM image of a pillar structured (75 nm diameter and 150 nm pitch) (**a**) silicon master and (**b**) transfer printed Au/Ti pillars

Although the lateral distortion does not occur on larger scales, it can still be possible that single structures might change their width/diameter resulting in a larger interdistance between them. Yet, if this takes place in the first replication step (negative copy of the master), the second replication (working stamp copy of the negative one) distortion would always be inverse to the first one, resulting in negligible changes of the lateral structure dimensions between working stamp and master. In Fig. 6, a comparison of (a) the master and (b) the final metal transfer is given. The diameters of the pillar structure just slightly increases from 73.4 ± 0.9 nm (silicon master) to 75.3 ± 1.6 nm (transfer printed metal) (Table 3). This increase is contributed to an expected conic growth of the deposited metal layers during the evaporation rather than a pillar diameter change during the replication process. Also, the measured increase in diameter by 1.9 nm is just close to the range of uncertainty of 1.8 nm.

**Table 3:** Diameter change

	Master	nTP
Diameter (nm)	73.4 ± 0.9	75.3 ± 1.6
Difference (nm)		1.9 ± 1.8 (+2.6 %)

The thickness of the metal film evaporated on the stamp was measured with an AFM and compared to the transferred metal structure height on the target substrate. In both cases, the same thickness of 20.3 nm was found within an uncertainty of ±0.7 nm. This proves a complete transfer of the metal from the stamp to the target substrate as well as a working anti-sticking layer, which was applied on the stamp surface (see Section “Methods”). Overall, shape retention is almost perfectly given.

### Large Area Transfer Print

**Fig. 7:**
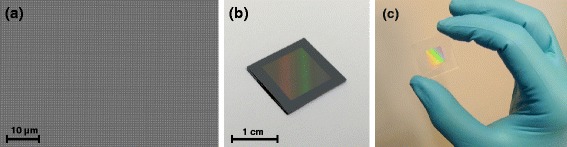
Large area nano-transfer print of 350-nm squares on a 1 ×1 cm^2^ area. (**a**) SEM image of part of the sample shown in **b**. (**b**) Picture of the whole nTP on a silicon substrate. (**c**) Picture of the nTP on a glass substrate

A great advantage of the nTP process presented in this paper is its more or less easy scalability towards large areas. To demonstrate this, we used a stamp with 350-nm squares, which were transferred over a comparably large area of 1 × 1 cm. Figure 7a shows a large area SEM image of the nTP on a scale, where single metal squares can still be distinguished. Characterized by SEM, the yield on the entire structured 1 cm^2^ area was measured to be above 99.9 %, where most of the imperfections were located almost exclusively near the edge of the patterned area.

The complete transfer is shown in Fig. 7b. Here, we first used silicon with native oxid as the target substrate. One can clearly see that the transfer is homogeneous and defect-free also over larger scales. To demonstrate that the same process can be successfully applied to other substrate types, a nTP was carried out on a standard borosilicate glass microscope slide (Fig. 7c). The response and reflection of light in the visible regime with the metal nanostructures indicate an effective transfer.

### Defect Tolerance and Master Lifetime

**Fig. 8:**
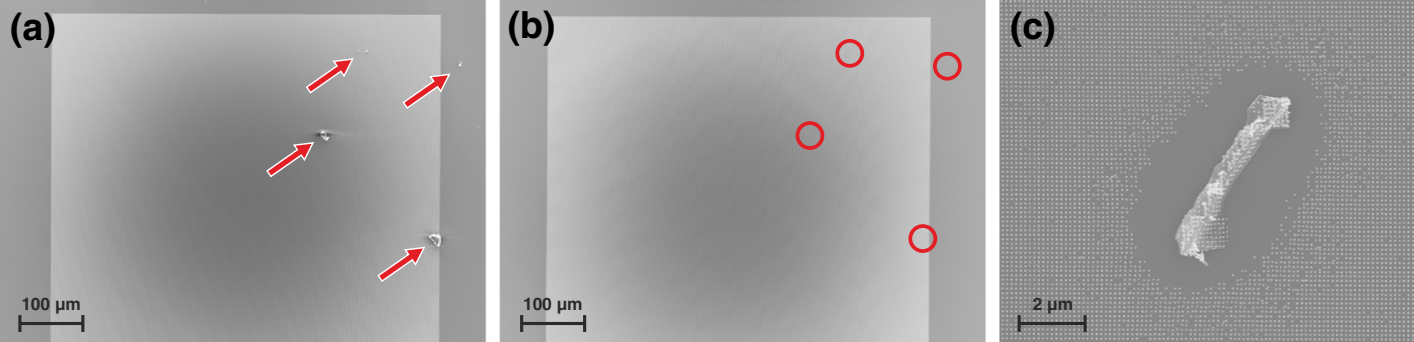
Comparison of a silicon master stamp (**a**) before an ormostamp replication with particle contaminations (marked by *red arrows*) and (**b**) self-cleaned surface of the same master after one OrmoStamp replication (**c**) nTP on a substrate with particle contaminations. Only in close vicinity of the contamination (some micrometers, depending on the particle size) no transfer of structures could be achieved

Even when working in a cleanroom, contamination of surfaces cannot be avoided completely. Already small particles can lead to a breakage of rigid stamps or substrates. The advantage of the here-used stamps made out of OrmoStamp®; are their semi-flexibility. While the polymer can sustain its high aspect ratio pattern on the nanometer scale without roof- or lateral collapse, it is still flexible enough on larger scales to overlay even micrometer-size particles. This defect tolerance leads to defect areas only in close vicinity of these contaminations (Fig. 8c), thus increasing the yield of the transfer print.

Unlike defects on the target substrates, defects on the silicon master will later be present on each working stamp and as a consequence also on the target substrate. Thus, cleaning of the silicon master stamp is required from time to time where the use of an ultrasonic bath or reactive chemicals might damage the fragile nanostructured surface.

Using OrmoStamp®;, there is a self-cleaning effect taking place during every replication procedure [14]. The liquid resin encapsulates any dust particles or residual resin during the drop casting which then are removed from the master surface in the demolding step. Figure 8a, b shows a nanopatterned silicon wafer with contaminations. After only one OrmoStamp®; treatment, the surface is completely particle free. With the process described here, stress on the silicon master is minimal since no pressure or temperature is applied during the master replication, thus extending the lifetime of the master dramatically.

## Conclusions

We have demonstrated nano-transfer printing with replicated semi-flexible, hybrid polymer stamps down to 40 nm feature size. Typical problems regarding nTP such as master reusability, defect tolerance, process cost and speed have been solved utilizing this replication technique. Working stamps based on OrmoStamp®; material are flexible enough to ensure intimate contact with the underlying substrate over large areas even in the presence of contaminating particles. Yet, the polymer is rigid enough for printing features in the sub-50-nm regime without lateral or roof collapse of the structures. Temperature and contact duration have been found to be the main parameters influencing the yield of nTP on silicon substrates. Using the optimized procedure presented in this work, a high yield above 99 % has been achieved repeatedly.

The main advantages compared to conventional working stamps made out of PDMS are the very high feature resolution and the more suitable UV-light curing process of the stamp material on the master mold instead of thermal curing, which results in a highly increased fabrication throughput.

Additionally, the avoidance of standard nanostructuring methods like e-beam and UV-light exposure, reactive ion etching as well as any kind of solvents or developers on the substrate during the nTP procedure make this process ideally suited for organic electronics where the organic materials tend to degrade rapidly under the influence of harsh conditions. In summary, this will open up opportunities for a wide range of applications of metal nanostructures in science and engineering such as energy conversion, sensing on solids and flexible substrates.

## Additional file

Additional file 1 Supporting information. (PDF 57 kb)
