# Supplementary material for: Large Area Nano-transfer Printing of Sub-50-nm Metal Nanostructures Using Low-cost Semi-flexible Hybrid Templates
Source: Nanoscale Res Lett. 2016 Mar 15;11:143. doi: 10.1186/s11671-016-1346-4 (PMC4791413; doi:10.1186/s11671-016-1346-4)
Supplement: Additional file 1 — Supporting information. (PDF 57 kb) [file 11671_2016_1346_MOESM1_ESM.pdf]

Supporting Information

Processes

If not otherwise specified in the main text, we used the following processes / parameters for the transfer printing experiments.

μm-transfer print with OrmoStamp replicas

**substrate**	p^+-type $< 100 >$ silicon with native oxide (SiMat)
**cleaning**	rinsing in acetone and isopropanol
**preconditioning**	5 min oxygen plasma (200 W, 60 Pa, 100 sccm O_2)
**metal evaporation**	e-beam PVD, Leybold 560, $P = 10^{-5}$ Pa.
	20 nm gold (0.2 nm/s), 3 nm titanium (0.1 nm/s)
**printing**	4 minutes, $200\,^\circ$C, 3 MPa (Obducat NIL)
**separation**	manually at approx. $160\,^\circ$C

nm-transfer print with OrmoStamp replicas (reference settings)

**substrate**	p^+-type $< 100 >$ silicon with native oxide (SiMat)
**cleaning**	rinsing in acetone and isopropanol
**preconditioning**	substrate: 6 min oxygen plasma (200 W, 60 Pa, 100 sccm O_2)
	stamp: 3 min oxygen plasma (200 W, 60 Pa, 100 sccm O_2)
**metal evaporation**	e-beam PVD, Leybold 560, $P = 10^{-5}$ Pa
	15 nm gold (0.3 nm/s), 3 nm titanium (0.1 nm/s)
**printing**	4 minutes, $200\,^\circ$C, 3 MPa (Obducat NIL)
**separation**	manually at approx. $160\,^\circ$C

Static water contact angle OrmoStamp

We conducted static contact angle measurements on OrmoStamp replica surfaces. We put a defined droplet of $5\,\mu$l of deionized water on the surface and captured a still photograph (see Figure S9). The efficacy of the "anti-sticking layer" treatment can be clearly observed as a significant increase in contact angle indicating a reduced surface energy of the stamp.

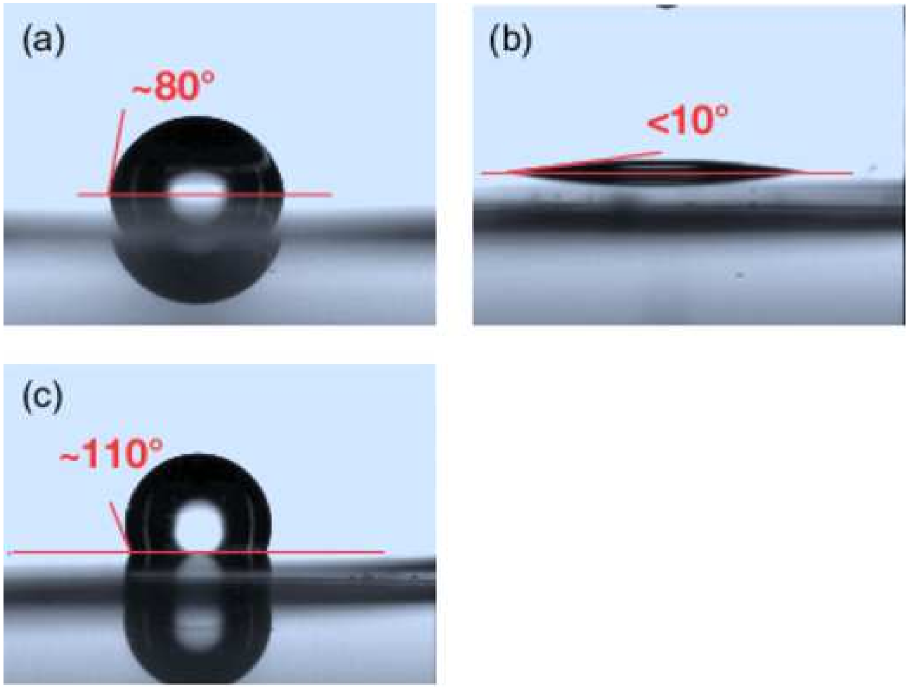

Figure S9 Static water contact angle on OrmoStamp surface: (a) as cured, (b) after mild oxygen plasma, (c) after physical vapor deposition of perflourooctlytrichlorsilane (PFOTS).

Transfer printed metal films

We characterized transfer printed metal films with atomic force microscopy in tapping mode. Typical image size was 7 μm x 7 μm. Gold/titanium films printed with OrmoStamp working stamps usually showed a root mean square (rms) roughness of $\sim$ 1 nm. Identical films transfer printed with PDMS stamps usually displayed a higher rms roughness between 3 nm and 6 nm. Figure S10 (a) and (b) show typical AFM images for metal films transferred with OrmoStamp and PDMS, respectively.

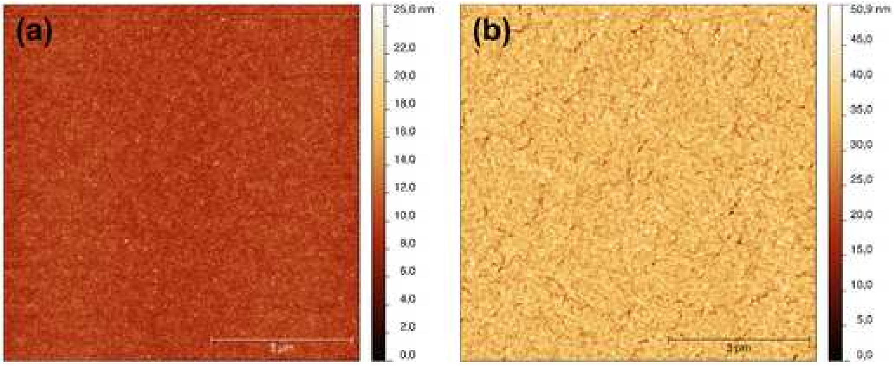

Figure S10 Thin Au/Ti film in Si, transfer printed with (a) OrmoStamp working stamp or (b) PDMS working stamp. RMS roughness is (a) $\sim$ 1.0 nm and (b) $\sim$ 3.1 nm.
